# Research of medical gases in Poland

**DOI:** 10.1186/2045-9912-3-17

**Published:** 2013-08-02

**Authors:** Robert P Ostrowski, Emanuela B Pucko

**Affiliations:** 1 Department of Experimental and Clinical Neuropathology, M. Mossakowski Medical Research Centre, Polish Academy of Sciences, 5 Pawińskiego St, 02-106 Warsaw, Poland

**Keywords:** Oxygen, Ozone, Gaseous transmitters, Volatile anesthetics, Noble gas, Poland

## Abstract

Research of medical gases is well established in Poland and has been marked with the foundation of several professional societies. Numerous academic centers including those dealing with hyperbaric and diving medicine conduct studies of medical gases, in vast majority supported with intramural funds. In general, Polish research of medical gases is very much clinical in nature, covering new applications and safety of medical gases in medicine; on the other hand there are several academic centers pursuing preclinical studies, and elaborating basic theories of gas physiology and mathematical modeling of gas exchange. What dominates is research dealing with oxygen and ozone as well as studies of anesthetic gases and their applications. Finally, several research directions involving noble gas, hydrogen and hydrogen sulfide for cell protection, only begin to gain recognition of basic scientists and clinicians. However, further developments require more monetary spending on research and clinical testing as well as formation of new collective bodies for coordinating efforts in this matter.

## Introduction

This paper summarizes and critically reviews research of medical gases in Poland done up to the year of 2012, predominately emphasizing interesting findings and promising directions to follow. We have searched bibliographical databases such as PubMed, MEDLINE, SCOPUS, Web of Knowledge and also used Polish search engines such as Expertus with publication records for employees of medical, agricultural, and military universities and research institutes in Poland. Timeline of these records stretched from April 1974 until January 2013. Of note, search terms denoting Polish affiliations were selected. In addition searching terms/phrases comprised hyperbaric oxygen, oxygen, ozone hydrogen, hydrogen sulfide, and carbon monoxide, nitric oxide, nitrous oxide, halothane, enflurane, isoflurane, sevoflurane and desflurane, and nitrogen. Then terms denoting noble gas were applied, including but not limited to helium and xenon followed by heliox and trimix, then pefluorocarbon and sulfur hexafluoride, such as in Sonovue. In general we focused on medical gases administered as exogenous agents, keeping in mind though that some of these gases can be formed endogenously and partake in human physiology. A few Polish review papers summarizing international research of different gases in medicine, including those with characteristics of gaseous transmitters, were included here as well. On numerous occasions we refer to original and review papers and commentaries written by internationally renowned experts of the subject.

### On the historical note

One alchemist from Poland can be considered a father of all Polish gas research. Michael Sendivogius, born in 1566 (died in 1636) studied philosophy, geometry, astronomy, mechanics, and alchemy at Cracow Academy of Poland and honed his skills at major European universities [1]. In 1604 he was able to obtain a gaseous substance that he named “food of life”, from heating salpetre, some 170 years before a major discovery of oxygen by Priestley. Apart from being an advisor to three Habsburg emperors - Rudolph II, Matthias and Ferdinand II, Sendivogius received hefty remunerations from the king of Poland Sigismundus III Vasa who had a confidence in him, and was an avid amateur alchemist himself.

During partitions of the country (since 1795) Polish nationals worked in the gas research at the universities annexed by Austria, Prussia, and Russia, producing great results. Those include an invention of new methods of liquefying oxygen and nitrogen by Professors Karol Olszewski and Zygmunt Wróblewski of the Jagiellonian University in Cracow. They were able to liquefy these gases first time in history from stable state air in 1883. Later Wróblewski as first published the data on hydrogen’s critical temperature, pressure, and boiling point (1885); both liquid oxygen and nitrogen have widespread use in medicine today [2-4]. In 1939, 21 years after regaining independence by Poland, the Institute for Maritime Medicine was erected in the coastal city of Gdynia which had started an era of maritime, diving and hyperbaric medicine. Since 1949 the Institute publishes a bulletin currently known as “International Maritime Health”, indexed in Medline. Currently the Institute is part of Medical University of Gdańsk as Inter-Faculty Institute of Maritime and Tropical Medicine [5]. It houses the National Centre for Hyperbaric Medicine and DAN Europe Office Polska located in Gdynia.

Polish research institutions conducting studies of medical gases include hyperbaric medicine centers, universities and institutes throughout the country.

Amongst organizations that nurture medical gas research, are the Polish Society of Anaesthesiology and Intensive Therapy issuing an official journal “Anaesthesiology Intensive Therapy” established 1968, while the Polish Hyperbaric Medicine and Technology Society, founded in 1997, since 2004 publishes “Polish Hyperbaric Research” journal, formerly distributed in a bulletin form.

### Oxygen in basic and clinical studies including hyperbaric research

The major achievement of Polish scientists are testing diagnostic applications of new parameters of oxygen physiology, thereby contributions to its understanding. As far as hyperbaric oxygen is concerned, Polish research not only indicates efficacy and clinical safety of this therapy in wound care and otorhinolaryngology, but novel mechanisms of treatment as well. It needs to be stressed however that interesting studies Polish of medical gas for diving medicine requires separate review.

Thus, one Polish group created a system based on neural network that can forecast arterial blood gas values computed from several input parameters including hemoglobin oxygen saturation [6]. Others elaborated purely mathematical model of oxygen gas exchange in the process of human respiration, that allows to examine gas exchange upon artificial oxygen ventilation and also to predict disturbances of oxygen exchange in pathological settings [7]. Polish scientists also adopted new oxygen status parameters known from literature such as oxygen extraction tension used e.g. to evaluate tissue hypoxia in diabetic children and adolescents [8].

Therapeutic action of oxygen has been reported in several studies advocating oxygen therapy at home, which improves life comfort and longevity of patients with lung diseases [9]. However in the field of neonatology it was found that oxygen increases the frequency of retinopathy of prematurity in neonates treated at FiO_2_ over 0.7 [10]. Another group has stated that oxygen should not exceed 40% during newborn resuscitation, and 100% oxygen is allowed only transiently [11].

In Poland it was commander Augustyn Dolatkowski, the first Polish member of Undersea & Hyperbaric Medical Society, who pioneered in lobbying for introducing hyperbaric oxygen into clinical practice. He postulated establishing at least three hyperbaric oxygen treatment centers, including one in district of Silesia, one in Warsaw and one on the coast [12]. Around that time Poland had operated oxygen chambers for patients in Sosnowiec (1971), Warsaw (1973) and Gdynia (1974) [13]. At the present time there are several more HBO chambers in the country including at Siemianowice Śląskie (est. 2002), Wrocław (2008), Łódź and Stalowa Wola (2009), and those opened in 2012: in Łęczna (eastern Poland), and another HBO center in Warsaw, run by a private clinic.

Clinical studies have shown that adjunct HBO, 10 × 2 ATA for 1 hour, is effective in the treatment of trophic ulcerations from venous insufficiency [14]. Interestingly, HBO at 2.5 ATA may improve thermoregulation by rearranging microcirculation in trophic ulcerations of the crura [15]. HBO administered within 2 to 60 exposures of 70 min. at 2.5 ATA was found to limit the extent of tissue lesions and extent of amputations in diabetic foot patients [16][17]. In larger group of patients with thermal burns HBO (2.5 ATA) reduced time to skin graft by 8 days, decreased tissue edema, risk of respiratory failure, and shortened duration of hospitalization [17]. HBO (2.5 ATA for 90 min) also proved to be an effective therapeutic adjuvant in Fournier’s gangrene, when given for seven days twice a day [18]. In patients with electric burns HBO (15–60 sessions) reduced infectious complications and overall mortality [19]. HBO (20 to 40 expositions per patient) cured postoperative sternal wound infection and mediastinitis in 55 patient enrolled in the clinical study [20]. Polish researchers as well found HBO beneficial for bacterial and to a lesser degree fungal otitis (14 to 45 exposures at above parameters) [21]. In other series of otorhinolaryngology cases HBO ameliorated hearing in 71% of patients under 18 treated for sudden sensorineural hearing loss; the authors used multiple HBO 2.5 ATA for 70 min. [22]. Furthermore, in adults 15 HBO sessions at 250 kPa (100% O_2_) alleviated chronic severe form of tinnitus compared to pharmacological therapy alone [23].

HBO also appears to be an effective adjunct treatment for radionecrotic type injuries including complications of neck irradiation [24]. Using thermographic approach others have found that HBO (30–60 × 90 minutes at 2.5 ATA) increases blood supply and tissue metabolism in cases of mandible osteoradionecrosis [25].

Polish preclinical HBO studies for neurological disorders aim to verify the mechanism of treatment against complex pathogenesis of neuron injury [26]. In one recent study authors demonstrated that both HBO and HBA (2.5 ATA for 60 min) increased neuronal survivability and functional performance after 5 min global cerebral ischemia in gerbils. Authors have postulated that equivalent reduction in cell death in both treatment groups can be attributed to a lowering effect of hyperbaria on brain temperature, regardless of air or oxygen [27]. The mechanisms of HBO have been also studied in the neonatal rats subjected to 60 min hypoxia-ischemia (HI). The authors demonstrated that HBO administered at 2.5 ATA at 1 or 3 hrs after HI reduced SOD activity in cerebral tissues while increasing GSH content and that HBO reduced brain damage more effectively than HBA treatment. Authors suggested that the production of free radicals was reduced with HBO and HBA, which manifested itself in decreased SOD activity [28]. When analyzing hypobaric hypoxia postconditioning the authors have found increased SOD activity and control GSH levels, which points towards distinctive molecular mechanisms triggered by each of the conditionining means.

### Ozone therapy

The major achievement of Polish ozone studies was to add novel clinical data allowing to broaden ozone indications in the clinical management of orthopedic surgery and dentistry, and demonstrate the application of ozone treatment in toxicology. In addition Polish researchers indicated which bacterial species are particularly susceptible to ozone therapy for dental care.

Similar to HBO, ozone inhibits septic processes in the wounds and facilitates wound healing; and it is believed that devising new methods of ozone administration may optimize those effects [29]. In this regard ozone was found to be prophylactic against septic complications in total hip plasty with and without cement [30]. Ozone administration was associated with completely cured bedsores in 16% of patients while in further 20% patients a significant improvement was noted as compared to the control group with traditional treatment [31]. As to lower extremities ailments ozone femoral artery injections administered for the treatment of atherosclerotic ischemia of the lower extremities more than doubled claudication distance [32]. Moreover, ozonated autohemotherapy (50 ug/ml) stimulated potentially beneficial changes in serum lipids including a decrease of total cholesterol and LDL levels in atherosclerotic ischemia of lower limbs [33]. Ozone (60 μg O3/mL i.v.) also normalized the activities of arylsulphatase, cathepsin D, acid phosphatase, and alpha-1-antitrypsin blood levels in patients with obliterative atheromatosis [34]. In gonarthrosis patients addition of ozone at 64 μg/m3 (0,032 ppm) in bath to the routine therapy reduced incidence of pain and diminished the use of analgesics [35].

Polish dental studies revealed that ozone kills number of viruses including HV1, HV2, and bacteria including *Actinobacillus actinomycetemcomitans* and *Campylobacter gracilis* and *sputorum* of the mouth and oral cavity; the bactericidal effects manifested themselves at 3,0 or 3,4 μg/mL ozone for 50–60 s [36,37]. Others have demonstrated potent oxidative effect of ozone towards *Streptococcus, Staphylococcus* and *Enterococcus* species isolated from the oral cavity [38]. While, ozone also decreases *Fusobacterium nucleatum* in gingival pockets [39]. The intracanal application of ozone proved to decrease periapical inflammation and caused absence of pain or exudate in root canal (HealOzone device, KaVo Co. up to 2100 ppm) [40]. Studies in 131 young patients age 11–12 revealed usefulness of ozone stopping progression of occlusal pit and fissure caries of premolar teeth with use of the device as above [41]. Moreover, in patients aged 21 till 65 ozone treatment was proposed for dentin hypersensitivity as a clinical method of eradicating bacteria from dentinal tubules thus facilitating beneficial penetration of fluoride, calcium, phosphates and tin minerals into the tooth in treatment (Heal Ozone, KaVo) [42].

On the other hand several Polish studies indicate a need for close monitoring over biochemical and proteomic indices of ozone therapy as it proved to have a decomposing impact on serum tocopherols (40–45 μl per 1 cm3 oxygen-ozone mixture in isotonic salt solution drip) [43], and oxidized blood lipids (30 μg/mL) [44]. Ozone however may have a therapeutic potential in toxicology; oxygen-ozone mixture injections reduced metallothionein level and cadmium accumulation in rat kidney after cadmium acetate intoxication [45]. An early bird for investigation of molecular mechanisms of ozone therapy was the proteomic study of ozonated blood by Ciborowski et al. [44]. The products of ozonation including ROS, H_2_O_2_ and LOPs play not only a role of biomarkers but are mediators of subcellular mechanisms of ozone treatment. Hydrogen peroxide activates tyrosine kinase which phosphorylates NF-κB fighting immune depression. Ozone generates LOPs which unlike ROS are longer lasting and activate endothelial cells leading to increased production of NO and vasodilatation, while may mediate bone marrow stem cells’ mobilization as well [46].

### Nitric oxide, carbon monoxide and hydrogen sulfide: gas giants and hot research spots

Nitric oxide, carbon monoxide and hydrogen sulfide belong to the gaseous transmitters. The gases in this group can be used therapeutically although except nitric oxide, have mostly investigational status at this point.

As for nitric oxide the established contributions of Polish researchers was to add new data on the clinical management of pulmonary hypertension and demonstrate its role oxide as gastroprotectant, and the involvement in nociception.

Inhalational nitric oxide has been a matter of investigation in cardiology of the adults and infants [47]. Patients with mitral valve and pulmonary hypertension inhaling NO (30 ppm) for 15 minutes before cardiac surgery procedures demonstrated favorable acute circulatory reaction, especially a reduction in pulmonary resistance [48]. Nitric oxide (8,15 ± 5,73 ppm) when inhaled upon imminent pulmonary hypertensive crisis after open heart correction in children caused a positive hemodynamic response manifested as reduced pulmonary arterial pressure and increased PaO_2_[49]. Indeed nitric oxide causes pulmonary arteries to display their vasodilatatory reserve as in cases of dilated cardiomyopathy [50].

Another research approach relies on the compounds releasing NO or triggering its production. Donors of NO, such as 3-morpholinosyndnoimine, S-nitroso-N-acetyl-DLpenicillamine, nitroglycerin and NO-aspirin have been demonstrated to act as gastroprotectants upon water immersion restraint stress [51]. Polish researchers found that NO mediated ulcer healing imparted by cholecystokinin [52]. In addition, endogenous NO has been found involved with the mechanisms of gastric preconditioning by means of 5 minute celiac artery occlusion [53]. It seems that the mechanism of NO mediated preconditioning can be similar to the one found in cardiac preconditioning and involve NOS, soluble guanyl cyclase and protein kinase G. Signal could be further transmitted to mitochondria via the opening of ATP-sensitive K+ channel and inhibition of the mitochondrial permeability transition pore in mucosal cells [54].

As to nociceptive action of NO, its endogenous donor L-arginine decreased antinociception effect of diazepam, chlordiazepoxide or clonazepam on the writhing test in mice consistent with the notion that excess NO produces hyperalgesia [55]. Additionally on the formalin test in rats, NO decreased spinal antinociceptive effect of clonidine but not that of baclofen [56].

Polish studies have also indicated benefits of carbon monoxide in organ transplant and preconditioning, beside studying mechanisms of toxicity upon excess CO [57]. One study has suggested that CO may be involved with the mechanisms of preconditioning, prior to organ transplant. The authors evaluated kidney transplant from CO poisoned donor and found prolonged tolerance of kidney to ischemia (total ischemia time was 24 hours and 35 minutes), effective urine production and uneventful clinical post transplant course. They postulated that CO may be responsible for preconditioning effect in clinical transplantation procedures, possibly due to anti-ischemic and anti-inflammatory properties of CO [58]. Indeed, CO was found to combat sepsis, mediate anti inflammatory effect in the liver and protects against renal ischemia-reperfusion injury [59]. Of note CO donor administration can inhibit thrombosis, through a decrease in active plasminogen activator inhibitor-1 or a decrease in fibrin generation, independently of guanyl cyclase (as in case of NO), which may prove beneficial with respect to organ transplant [60]. As reviewed by Amano et al. molecular mechanism of CO at therapeutic doses may involve modulating immune response in graft donors, in particular diminishing expression of MHC class II on dendritic cells and reducing APC –induced T cell proliferation, TNF-α and IFN-γ, and IL-2 production [61]. Furthermore CO increases HIF-1α and VEGF expression as well as bcl-2 production leading to reduced apoptosis.

H_2_S is a gaseous neurotransmitter involved with long-term potentiation and smooth muscle relaxation [62]. In the living organisms H_2_S forms an aqueous solution of weak acid or is present as an anion. Although administration of gaseous H_2_S has been used in research, its proper dosage appears challenging (usual range 20-80 ppm) [63].

In the field of hydrogen sulfide studies Wiliński and colleagues at the Jagiellonian University have shown the immense impact of a variety of pharmacological agents on endogenous H_2_S production and postulated the pharmacomodulatory role of H_2_S in this setting [64]. According to their investigations Vitamin D3 at doses up to 40000 UI/kg b.w. increased tissue free H_2_S concentration in the heart (by 74.1%) and to a lesser degree in the brains and kidneys of male SJL mice [65]. The authors further demonstrated that other repeatedly administered drugs, including aspirine, acetaminophen, atorvastatin, ramipril, amlodipine, digoxin and and carvedilol, affect sulfur metabolism and H_2_S [65]. Interestingly they found that aspirin (10 mg/kg b.w.) decreased H_2_S levels in the brain of female albino Swiss mice (although increased in the liver) contrary to some other reports showing aspirin-induced H_2_S elevation in female BALB/c and male B10.PL mice. Therefore, they postulated that H_2_S production in mouse brain could be strain dependent [66]. Acetaminophen (30 mg/kg b.w.) produced marked increase in H_2_S tissue concentration in the heart and kidney while decreased its levels in brains of female CBA mice [67]. In same strain of mice the effect of atorvastatin injected at a dose of 5 or 20 mg/kg, remarkably increased H_2_S concentration in the kidney, and also in the brain and heart at a dose of 5mg/kg, while decreased in the liver at both doses [68]. In CBA mice of both sexes, Ramipril, 5 or 10 mg/kg increased H_2_S tissue concentration in mouse liver while decreased in kidney, where H_2_S by itself has been identified as inhibiting ACE [64]. Experiments with cardiological medications showed that carvedilol at high doses increased tissue H_2_S level in the heart (by 75%) kidney and liver however decreased in the brain of SJL female mice. Digoxin increased H_2_S in all investigated organs (BALB/c male mice), especially in the heart (by 22%) while amlodipine tended to decrease it in all examined organs (CBA mice) [69-71]. Overall these findings further implicate the H_2_S in the nociception, NO and CO metabolism, ACE regulation, and cardioprotection, however, with little indication as to the molecular mechanisms downstream of H_2_S. Studies by Calvert et al. have shown that H_2_S is a potent inducer of Nrf2 which governs a group of antioxidant and drug metabolizing genes [72]. How Nrf2 activation relates to findings by Wilinski et al. awaits further investigation.

Above mentioned gaseous compounds have been proposed as therapeutic agents, e.g. the administration of H_2_S donors for renal diseases, associated with deficiency of endogenous H_2_S [73]. Since NO can increase red blood cell deformability, its donors have been proposed as agents improving blood fluidity [74]. However any carbon monoxide treatment due to a risk of toxicity would need to be closely monitored [75].

### Volatile anesthetics in basic and clinical research

Polish researchers made valuable contributions to pros and cons discussion over advantages of intravenous vs. inhalational anesthesia. They increased overall number of clinical cases examined in the world literature in order to establish superiority of anesthetic methods in this regard. The achievement worth emphasizing was also to demonstrate the involvement of endogenous opioids in the mechanisms of volatile anesthesia/analgesia. The work by Polish researchers on EEG pattern analysis furthered the understanding of mechanisms behind inhalational anesthesia. Time will tell whether new algorithms of EEG analysis elaborated in Poland will be broadly implemented for anesthesiological procedures.

The Polish researchers were also amongst the first to show advantages of newer halogenated gases over older agents e.g. they showed that isoflurane has lower genotoxicity than halothane towards peripheral blood lymphocytes [76]. Other authors demonstrated that compared to isoflurane, enflurane produces stronger alterations in the activities of brain enzymes responsible for catabolic processes, and the rates of exchange and transport of energetic substrates in the striatum and hypothalamus in the rabbit brain. Therefore, the authors suggested that enflurane offers lower safety than isoflurane [77]. Polish authors also conducted the comparisons between different 3rd generation agents e.g. sevoflurane and isoflurane (0.6-1.5 MAC) for cardiopulmonary bypass, by assessing total usage of phenylephrin administered against drops in arterial pressure, which together with hemodynamics data revealed no difference in major parameters including cardiopulmonary bypass and aortic cross clamp times, ejection fraction, temperature, and EuroSCORE indexes. [78]. Researchers at Medical University of Lublin found that desflurane increased peripheral perfusion in elective gynecologic surgery patients as determined with perfusion index [79]. The classic advantage of desflurane, rapid emergence from anesthesia, was studied in obese patients by Dr. Gaszynski who found faster recovery and faster (16,3 vs 22,4 min) reaching 10 points on the Aldrete scale by those patients (47 individuals with BMI >40 kg/m2; ASA 2) anesthetized with desflurane for gastric banding, compared with sevoflurane [80]. Interestingly, sevoflurane anesthesia can reduce rocuronium requirement as sevoflurane potentiates neuromuscular block [81]. Similarly sevoflurane decreased the required dose of muscle relaxant in laparoscopic gastric banding [82].

Other comparisons were made between halogenated anesthetics such as isoflurane and injectable anesthetics e.g. propofol. Both isoflurane and propofol provided effective clinical anesthesia and could be safely used for short gynecological surgery. Times to eye opening, to recovery of spontaneous breathing, and to extubation were equivalent between inhalation and injection anesthesia groups [83]. Similarly, single-breath vital capacity rapid induction with 8% sevoflurane followed by 1.5% sevoflurane with N_2_O was as effective as propofol for short gynecological procedures [84]. Desflurane and propofol combined with thoracic epidural anesthesia showed similar effectiveness and safety profile in one lung ventilation upon lobectomy [85]. As demonstrated in patients with embolization of cerebral haemangiomas or arteriovenous malformations, sevoflurane provides safe and stable anesthesia for interventional neuroradiological procedures compared to propofol/fentanyl anesthesia [86].

However quite a few Polish studies investigated the very mechanism of anesthetic action of halogenated gases both on basic science and clinical grounds where the understanding is far from complete. These studies showed that once postulated theory of anesthesia, referring to endogenous opioid peptides may appear plausible as leu-enkephalin has been found increased in hypothalamus, hippocampus, mesencephalon and lumbar segment of spinal cord in isoflurane anesthetized rabbits. However both leu-enkephalin and met-enkephalin decreased in the cervical segment while met-enkephalin content dropped in thoracic part of the spinal cord. Unlike isoflurane halothane decreased leu-enkephalin content in the hypothalamus, while increased in the hippocampus. Methionine-enkephalin levels however increased upon halothane and persisted at least 1 hr after anesthesia has been ceased [87,88].

Polish researchers actively participate in devising new analysis modes for investigating mechanisms and safety of volatile anesthetics. BIS monitoring is commonly used for studies aimed at optimization of anesthesia for particular clinical procedures. With this approach researchers found e.g. that desflurane allows to wake faster, when compared with sevoflurane and isoflurane, after lumbar laminotomy in 60 adult patients [89]. One Silesian group studied EEG propagation under sevoflurane using directed transfer function method. Indeed the assessment of propagation directions of EEG signals between channels may upgrade analysis of spatial distribution of EEG activity [90]. Furthermore, quantitative analysis of EEG for transition from consciousness to unconsciousness for isoflurane, sevoflurane and desflurane revealed relative powers of particular EEG waves [91]. As to desflurane, researchers further elaborated new methods to detect sharp wave and slow wave components of the EEG pattern to investigate of possible side effects of this anesthetic gas in the clinical settings [92].

Notably the impact of inhalational anesthetic agents on body systems/organs has been studied in Poland as well. Studies have indicated that sevoflurane low flow (0.8–1 L min–1) air-oxygen-sevoflurane (1–3 MAC) does not increase nephrotoxicity of chemotherapeutic agents [93]. It also turned out that both isoflurane and halothane could affect airway transepithelial potential difference in the isolated rabbit tracheal wall, which suggests depressing airway clearance. Notably, isoflurane distinctly less affected the depolarization component while the reaction to both gases was fully reversible [94]. In the meantime studies directed at risk of toxicity turned out quite favorable for sevoflurane, which did not induce any increase in DNA migration in blood lymphocytes proliferating in vitro, in contrast to halothane and isoflurane [95]. However sevoflurane’s impact on liver function has been reconfirmed. Sevoflurane was found to be a more potent microsome liver cytochrome b5 inhibitor than desflurane [96].

Then the mechanisms of brain and heart protection with volatile anesthetic agents gained Polish researchers’ interest as well. As stressed in one original paper by Baranowska et al. neurological complication during heart surgery relate to long duration of ventilation, prolonged crossclamping and CPB while low CPP, history of atrial fibrillation or stroke as well as advanced age, hence the need for clinically effective preconditioning procedures [97]. Both isoflurane and sevoflurane could reduce biochemical markers of brain injury including MMP-9, GFAP and S-100β, and ameliorated disturbed arteriovenous magnesium concentration differences in patients after CABG, as compared to intravenous anesthesia [98,99]. Studies conducted in USA and elsewhere have shown multiple mechanisms for brain protection with isoflurane including activation of ATP-regulated potassium channels, upregulation of NOS and PI3K-AKT and reduced excitotoxic stress [100]. Recent studies have indicated that major mechanism of isoflurane-induced neuroprotection leads through sphingosine-1-phosphate/phosphatidylinositol-3-kinase/Akt pathway [101].

In addition, Polish experimental studies have shown that sevoflurane inhibits synthesis and release of excitatory neurotransmitters, including glutamate, and inhibits activation of microglia and astrocytes responsible for the occurrence of inflammation after ICH [102,103].

Some Polish researchers have examined volatile anesthesia against cardiac injury as well. One study has found that anesthetic preconditioning with sevoflurane (20 minutes of sevoflurane 2 vol% inhalation at 10 min prior to CABG under CPB) acutely activates ROS generation in coronary sinus blood, but then decreases blood levels of interleukin-6 at six hours after surgery [104].

Particularly interesting direction in research is formed by nitrous oxide studies. N_2_O is commonly used to decrease anesthetic requirements of halogenated gases allowing to study different anesthetic agents at lower concentrations. Upon such studies Polish researchers found that children anesthetized with N_2_O/O_2_–fentanyl had higher mivacurium requirement than those in halogenated anesthetics/N_2_O/O_2_ group, which may carry implications for residual muscle weakness [105]. The duration of intubating dose and spontaneous recovery of neuromuscular transmission were significantly prolonged by halogenated gases as compared to N_2_O/O_2_ –fentanyl anesthesia [106].

Also, encouraging results were reported for nitrous oxide in sedation. Some Polish authors postulated that nitrous oxide should be wider used for sedation in oral surgery [107]. One study has found however that despite good outcomes of nitrous oxide sedation for dental treatment 6 out of 20 children refused to breathe through the mask under any circumstances and had to be subjected to general anesthesia [108]. Definitely, caution should be advocated for the use of N_2_O or halogenated anesthetics in sedation especially in children as there is a substantial body of data pointing towards possible neurodegenerative effects of those in case of prolonged exposure [109].

### Noble gas in Polish medical research

Amongst noble gases helium has been studied quite extensively in the intensive care and diving medicine in Poland. Polish scientists contributed to establishing usefulness of breathing helium for diving and treatment of diving accidents, and used against neonatal respiratory distress as well. These series of diving cases are of unique value, especially diving accidents cannot be easily replicated. Helium neonatal studies also have a great importance for clinical practice. These have proved that medical gas research conducted in Poland can advance to clinical trials registered with NIH.

Helium in diver’s breathing gas allows to decrease partial pressure of nitrogen thus diminishing unfavorable effects of the latter, helium also allows to decrease the work of breathing. In addition heliox, which is a mix of helium and oxygen, has been proposed to counter unfavorable hematological consequences of diving, amongst which loss of circulating platelets appears most common [110]. With this regard, breathing trimix (O2 – 18.5%, N2 - 44%, He – 37.5%) prevented reduction in the number of platelets and loss of coagulation factor XII in male professional divers subjected to 0.7 MPa exposure (35 min. plateau). Since solubility and diffusion coefficients of helium are relatively low there will be faster release of dissolved gas during decompression and reduced formation of gas bubbles in diver’s blood, hence reduced number of platelet aggregates [111].

Breathing gas mix with helium has been investigated clinically as well. As reviewed by Polish authors, heliox, possess suitable properties as a carrier of aerosol therapy and a breathing mix upon mechanical ventilation. These include facilitation of laminar flow and reduction of airway resistance. Therefore it has been postulated that patients with severe bronchospastic diseases may experience the greatest benefits of breathing heliox [112]. Others have postulated that heliox containing 20% oxygen and 80% helium seems to be a very promising treatment in the management of the acute phase of severe respiratory disturbances in the newborn [113]. A group of researchers at Poznań University of Medical Sciences administered heliox through mechanical ventilation in the meconium aspiration syndrome in neonates. This particular study was a subject to a clinical trial registered at http://clinicaltrials.gov. Recently the authors have reported that there was a significant increase in the PaO(2)FiO(2) ratio during heliox ventilation in their clinical setting [114]. As to moleculaer mechanisms helium imparts influence on molecular machinery of the cells within lungs, the immune system, and the blood, besides improving work of breathing. At least part of therapeutic effects of helium may be ascribed to activation of mitochondrial ATP regulated potassium channels for cell protection [115].

### On some gaseous compounds for ultrasonography

It is an important contribution of Polish researchers to prove safety of ultrasound sulfur hexafluoride microbubble contrast agent (SonoVue) with respect to hematologic system. Furthermore, based on extensive research Polish authors postulated that contrast enhanced ultrasound may become a method of choice for determination of character of liver lesion.

Micro bubble contrast agents, formulated with encapsulated gaseous compounds, have received researchers attention for the studies of contrast enhanced ultrasound (CEUS). In the beginning ultrasound contrast agents utilized air microbubbles (generated via sonication) or galactose microparticles [116]. Later on, many studies have investigated the ultrasound second-generation contrast agents such as SonoVue. This particular agent is formed by stabilized microbubbles of sulfur hexafluoride (SF_6_) gas and exhibits an outstanding safety profile [117]. Bubbles of SF_6_ are extremely small; the bubble diameter in SonoVue averages 2.5 μl, which allows to minimize total volume of injected gas [118]. Polish researchers elaborated on SonoVue safety by demonstrating with electrophoretic mobility techniques that SonoVue did not produce large changes with respect to surface charge density of red blood cell membranes [119]. Based on the results obtained in Polish academic hospitals contrast enhanced ultrasound has been postulated as a modality of choice in the diagnostics and differentiation of benign focal liver lesions [120]. Although being less accurate than histological assessment CEUS still allows to predict malignant character of renal lesions [121]. CEUS has been also compared to CT with respect to accuracy of imaging. Contrast-enhanced ultrasonography may be considered an alternative technique to CTA in monitoring patients after abdominal aortic aneurysm repair [122]. There was a high accordance between CEUS and CT in the diagnostics of metastatic liver lesions [123]. In cases of liver hemangiomas CEUS managed to approximate CT accuracy, however specificity and negative predictive value appeared lower [124].

### Concluding remarks and prospects of researching medical gases in Poland

In summary research of medical gas in Poland is in large part directed at testing novel clinical applications of medical gases. However thorough investigations with the use of molecular biology and genetics techniques, including omics approach, are developing as well. Undeniably, in order to progress, research of medical gas needs to be financed from major dedicated funds. This appears necessary, especially to assure diversity in translational research, but also to integrate medical gas research with stem cell and molecular biology investigations. Also, to further medical gas research in Poland, international collaborations with leading medical gas research centers are warranted, for mutual benefits.

## Abbreviations

ACE: Angiotensin-converting-enzyme; ATA: Atmosphere absolute; BIS: Bispectral index; CABG: Coronary artery bypass grafting; CEUS: Contrast enhanced ultrasound; CO: Carbon monoxide; CPB: Cardiopulmonary bypass; CPP: Cerebral perfusion pressure; CT: Computed tomography; FiO2: Fraction of inspired oxygen; GFAP: Glial fibrillary acidic protein; GSH: Glutathione; H2O2: Hydrogen peroxide; H2S: Hydrogen sulfide; HBA: Hyperbaric air; HBO: Hyperbaric oxygen; HI: Hypoxia-ischemia; HIF-1α: Hypoxia inducible factor 1α; HV1: Herpes virus type I; ICH: Intracerebral hemorrhage; ICP: Intracranial pressure; ICV: Intracerebroventricularly; LDL: Low-density lipoprotein; LOPs: Lipid oxidation products; IFN-γ: Interferon γ; IL-2: Interleukin 2; MAC: Minimum alveolar concentration; MMP-9: Matrix metalloproteinase-9; MHC: Major histocompatibility complex; N2O: Nitrous oxide; NF-κB: Nuclear factor kappa-light-chain-enhancer of activated B cells; NO: Nitric oxide; Nrf2: Nuclear factor E2–related factor; PaO2: Partial pressure of oxygen in the plasma of arterial blood; Ppm: Parts per million; ROS: Reactive oxygen species; SF6: Sulfur hexafluoride; SOD: Superoxide dismutase; VEGF: Vascular endothelial growth factor.

## Competing interests

The authors declare that they have no competing interests.

## Authors’ contributions

RPO: drafted the manuscript. EP: was involved in drafting the manuscript. Both authors read and approved the final manuscript.
